# Road traffic noise and cognitive function in older adults: a cross-sectional investigation of The Irish Longitudinal Study on Ageing

**DOI:** 10.1186/s12889-021-11853-y

**Published:** 2021-10-08

**Authors:** Ciarán Mac Domhnaill, Owen Douglas, Seán Lyons, Enda Murphy, Anne Nolan

**Affiliations:** 1 Economic and Social Research Institute, Dublin, Ireland; 2 Department of Economics, Trinity College Dublin, Dublin, Ireland; 3 School of Architecture, Planning and Environmental Policy, University College Dublin, Dublin, Ireland; 4 Eastern and Midland Regional Assembly, Dublin, Ireland; 5 The Irish Longitudinal Study on Ageing, Trinity College Dublin, Dublin, Ireland

**Keywords:** Road traffic noise, CNOSSOS-EU, Health, Cognitive function, Older adults, Ireland, Air pollution

## Abstract

**Background:**

The World Health Organization published updated Environmental Noise Guidelines in 2018. Included are recommended limit values for environmental noise exposure based on systematic reviews for a range of health outcomes, including cognitive impairment. There is emerging evidence in the literature that chronic exposure to road traffic noise may affect cognitive function in older adults, but this relationship is not well established. This study spatially linked nationally representative health microdata from The Irish Longitudinal Study on Ageing to building-level modelled noise data for two cities in the Republic of Ireland. This was used to investigate associations between exposure to road traffic noise and cognitive function in a sample of older adults, independent of a range of socio-demographic and behavioural characteristics, as well as exposure to air pollution.

**Methods:**

We used the Predictor-LimA Advanced V2019.02 software package to estimate noise originating from road traffic for the cities of Dublin and Cork in Ireland according to the new common noise assessment methodology for the European Union (CNOSSOS-EU). Noise exposure values were calculated for each building and spatially linked with geo-coded TILDA microdata for 1706 individuals aged 54 and over in the two cities. Ordinary least squares linear regression models were estimated for eight standardised cognitive tests including noise exposure as an independent variable, with standard errors clustered at the household level. Models were adjusted for individual sociodemographic, behavioural and environmental characteristics.

**Results:**

We find some evidence that road traffic noise exposure is negatively associated with executive function, as measured by the Animal Naming Test, among our sample of older adults. This association appears to be accounted for by exposure to air pollution when focusing on a sub-sample. We do not find evidence of an association between noise exposure and memory or processing speed.

**Conclusions:**

Long term exposure to road traffic noise may be negatively associated with executive function among older adults.

**Supplementary Information:**

The online version contains supplementary material available at 10.1186/s12889-021-11853-y.

## Background

In the European Union, Strategic Noise Mapping (SNM) and noise action planning is undertaken according to the EU Environmental Noise Directive 2002/49/EC (END), as amended by EU Directive 2015/996/EC. The primary aim of the END, and the legal obligation of EU Member States to engage in the SNM process, is to define a common approach intended to avoid, prevent or reduce on a prioritised basis the harmful effects resulting from exposure to environmental noise. As such, noise assessment is increasingly important as the extent of empirical evidence linking nonauditory negative health outcomes to transportation noise exposure is growing considerably [1]. Noise mapping in the EU Directive is a process which has two strands – first, the creation of contour maps which are based on noise calculation along uniform grids; and second, the estimation of population exposure which is typically based on separate modelling of noise at the external façades of buildings (i.e. a receiver point is placed at the façade of each building). L_den_ and L_night_ are EU (A-weighted) indicators of annual average noise levels developed specifically for the END. L_den_ is a descriptor of noise levels based on energy equivalent noise level (L_eq_) over a whole day with a penalty of 10 dB(A) for night-time noise (23.00–7.00) and an additional penalty of 5 dB(A) for evening noise (i.e. 19.00–23.00). L_night_ is the corresponding indicator for the night-time period. They are key indicators used in the Strategic Noise Mapping (SNM) process and for estimating population exposure to environmental noise.

The World Health Organization (WHO) published updated Environmental Noise Guidelines for the European Region in 2018 [2] which provide recommended noise limits for road, rail and aircraft using the L_den_ and L_night_ indicators for exposure at the most exposed building façade outdoors. These noise limit values are based on an assessment of evidence resulting from systematic literature reviews for associations between environmental noise exposure and cardiovascular and metabolic effects [3]; annoyance [4]; effects on sleep [5]; cognition [6]; hearing and tinnitus [7]; adverse birth outcomes [8]; and quality of life, mental health and wellbeing [9]. In Europe, one in five people are exposed to levels of environmental noise that are considered harmful to health [10].

A systematic review on environmental noise and effects on cognition was undertaken by Clark and Paunovic [6] and included quantitative nonexperimental studies published up to June 2015. As noted by Douglas and Murphy [11], 34 studies were included in the Clark and Paunovic [6] review, all of which were of child populations. Evidence pertaining to older persons is much more limited, despite older persons being identified as a vulnerable group for noise exposure [10, 12]. Since 2015, a number of studies have emerged which have investigated associations between noise exposure and cognitive impairment in older adults, but results have been mixed. Furthermore, the importance of simultaneously investigating exposure to noise and air pollution has been highlighted in the literature [11, 13, 14]. An updated systematic review, including evidence published up to 2019, was conducted by Clark et al. [15].

The objective of this study is to model noise exposure and to investigate associations between exposure to road traffic noise and cognitive function in a sample of older adults, independent of a range of socio-demographic, behavioural and environmental characteristics. We focus on chronic noise exposure in this study, with cognitive tests carried out in a standardised format to separate any effect of chronic noise from acute noise. By combining road traffic noise data modelled using the new CNOSSOS-EU methodology for receiver points at all buildings in the agglomerations of the two largest cities in the Republic of Ireland, Dublin and Cork, with geo-coded microdata on the health and wellbeing of older adults from The Irish Longitudinal Study on Ageing (TILDA), we aim to broaden the evidence base on relationships between environmental noise exposure and cognitive health among older adults.

Recent studies indicate that exposure to air and noise pollution from traffic sources may be associated with cognitive impairment [14]. Most epidemiologic studies have focused on the association between air pollution and cognition function, with the association between noise and cognitive impairment far less studied [16], particularly for older people [17]. In recent years, a number of studies have been published which have explored this relationship.

A 2015 review of evidence by Tzivian et al. [14] noted that associations between noise and neurocognitive function remained under-investigated. Mild cognitive impairment is defined as a ‘cognitive decline greater than expected for an individual’s age and educational level, but that does not interfere notably with activities of daily life’, and is regarded as a major risk factor for progression to Alzheimer’s disease. Using cross-sectional data from the Heinz Nixdorf Recall study in Germany, Tzivian et al. [18] identified positive associations between road traffic noise exposure and the probability of a mild cognitive impairment diagnosis. The associations with road traffic noise were robust to adjustment for air pollution, suggesting worse cognitive function in populations exposed to higher noise levels. A subsequent cross-sectional study further explored the combined effect of co-exposure to road traffic noise and air pollution, and suggested that air pollution and road traffic noise may act ‘synergistically’ to affect cognitive function in adults, as stronger negative effects were associated with a combined exposure to both stressors than would be implied by an addition of the effects associated with each stressor in isolation [13]. It is worth noting that exposure to each noise and air pollution may affect cognition through different mechanisms, and that each exposure may thus have separate effects on cognition. Noise exposure has been hypothesised to affect health indirectly through an impact on stress or sleep [6], while proposed mechanisms through which exposure to air pollution may affect cognition include, for example, the impact of air pollution on the circulatory system [19, 20].

In a retrospective cohort study using primary care data for 130,988 adults aged 50–79 in London in 2005, Carey et al. [21] found a positive exposure response relationship between dementia and most measures of air pollution. They found that adults living in areas with the highest fifth of NO_2_ concentration (> 41.5 μg m^− 3^) versus the lowest fifth (< 31.9 μg m^− 3^) were at a higher risk of dementia. Increases in dementia risk were observed with PM_2.5_ and L_night_, but only NO_2_ and PM_2.5_ remained statistically significant in multi-pollutant models.

In their 2019 study, Fuks et al. [17] investigated the association between residential exposure to road traffic noise and cognitive function in a cohort of 288 elderly women. Data was drawn from the German Longitudinal Study on the Influence of Air Pollution on Lung Function, Inflammation and Aging. Cognitive function was assessed using the Consortium to Establish a Registry on Alzheimer’s Disease (CERAD-Plus) Neuropsychological Assessment Battery. CERAD-Plus consists of eighteen test items belonging to four cognitive domains (semantic memory, episodic memory, constructional praxis and executive function) and the Mini Mental State Examination (MMSE). No associations were identified between night-time noise exposure and cognitive scores in the main model. Adjusting for air pollution revealed a weak association with an improved score on a trail making test of executive function, although the statistical power to detect associations was constrained by a relatively small sample size.

Finally, in their 10-year follow-up study of a cohort of 1612 Mexican American participants from the Sacramento Area Latino Study on Aging (SALSA), Yu et al. [16] found that the risk of dementia/CIND was elevated when 24-h and night-time noise were higher than 75 and 65 dB respectively.

While not exploring noise exposure specifically, other studies have investigated the potential effects of living near major roadways on cognition. One prospective cohort study identified an association between such proximity and poorer performance in tests of verbal learning and memory, psychomotor speed, language and executive function among older adults [22]. In another cohort study, residential proximity to a major roadway was found to be linked with increased incidence of dementia among adults, a relationship only partly accounted for by exposure to air pollution. The authors suggested that this points to the potential independent influence of road traffic noise [23]. Indeed, a study using time series data on healthcare demand identified a relationship between acute road traffic noise and healthcare demand related specifically to Parkinson’s disease, independent of air pollution [24].

The literature has also examined exposure to other sources of environmental noise such as railway and aircraft noise. Increased attentional lapses were identified in adults living near a railway in one laboratory study based in France [25], while attending a school located near a major airport was found to be associated with impaired memory in children in two other European prospective cohort studies [26, 27]. These findings suggest that potential associations between road traffic noise and various domains of cognitive function should be examined. A distinction should also be drawn between acute and chronic exposure to environmental noise. The impact of acute noise in occupational settings on cognitive performance has received some attention in the literature [28, 29]. It has been suggested, however, that while acute noise may have marginal effects on cognitive function, chronic exposure could have far more detrimental effects [25].

While the evidence from epidemiologic studies remains inconsistent, Yu et al. [16] reported how animal studies have linked noise exposure to decreased cognitive performance. Experimental studies suggested that noise acts as a stressor that can reduce brain structures that are integral to mediating stress responses [30, 31]. Other studies suggested that noise stressors can ultimately lead to a dysregulation of the prefrontal cortex responsible for cognitive abilities, including executive function [32, 33]. Furthermore, noise may also activate the hypothalamic-pituitary-adrenal axis [34–37], which could contribute to metabolic dysregulation [37–40] and cognitive damage. A 2017 animal study by Guo et al. [41] also suggested the fluctuation of stress-responses as one possible mechanism through which noise exposure may affect cognitive function.

## Methods

We estimated levels of exposure to road traffic noise for receivers positioned at the exposed facades of all buildings in the cities of Dublin and Cork according to the common framework for strategic noise mapping and population exposure estimation developed for EU Member States, CNOSSOS-EU [42]. The CNOSSOS-EU model has been validated for low-to medium and medium-to-high traffic flow on roads in the Dublin agglomeration by Murphy et al. [43]. Their results show that the CNOSSOS-EU model converges closely with roadside measurements using calibrated sound level meters (SLMs) – within 0.1–0.6 dB(A) for low-to-medium and 0.2–2.0 dB(A)) for medium-to-high traffic flow respectively. For medium-to-high traffic, the validation was undertaken during a weekday period from 15:15–16:15 and included comparing modelled results with those from 10 Type 1 SLMs and one Type 2 SLM. For low-to-medium traffic, the validation was undertaken during a weekday period from 13:00–14:00 and included comparing modelled results with those from 4 Type 1 SLMs. This modelled noise exposure data was spatially linked with geo-coded microdata on health, wellbeing and socio-economic circumstances from TILDA at the building scale, thus assigning a building-specific estimated level of noise exposure to each respondent at their residence. Data linkage was conducted using a Geographic Information System (GIS) platform, QGIS 3. Regression models were applied to various cognitive outcomes to identify and measure relationships with noise exposure, adjusting for a range of potential confounding socioeconomic and behavioural factors as well as for air pollution. Statistical analysis was conducted using Stata 14.

### Study population

We relied on data from The Irish Longitudinal Study on Ageing (TILDA) [44, 45] for our study population. TILDA is a nationally representative longitudinal study of over 8000 people aged 50 and over in Ireland, which collects information on all aspects of health, economic and social circumstances. All members of sample households who are aged 50 and over are surveyed. TILDA is harmonised with the Survey of Health, Ageing and Retirement in Europe, the English Longitudinal Study of Ageing, the Health and Retirement Survey (HRS) and the HRS international network of studies. The geo-code of each TILDA respondent’s home address is recorded, allowing the dataset to be linked with other geo-coded spatial data. Data collection for the third wave of the survey, which was employed in this paper, was carried out between March 2014 and October 2015 on 6396 individuals aged 54 and over.

Three different methods of data collection are used by TILDA. First, trained interviewers conduct Computer Assisted Personal Interviewing (CAPI) in each respondent’s home. Second, a self-completed questionnaire that captures more potentially sensitive data is filled out and returned by post. Finally, respondents are invited to attend a nurse-led health assessment at specialised Health Assessment Centres, or a modified partial assessment in their homes if travel to a centre is not practicable [32].

We used data gathered from all three stages in this study. We matched data on estimated noise exposure to respondents living in Dublin and Cork, giving a sample size of 1706 for analysis. A flow chart detailing the sample size is provided in Appendix A (see Additional File 1). This sample size varied by question due to varying rates of non-response. Further details on TILDA are outlined by Kearney et al. [45]. Twelve TILDA respondents who are exposed to railway [46] or aircraft noise [47] above 45 dB at night were removed from the analysis to further isolate any associations between road traffic noise specifically and cognition.

### Outcomes: cognitive health variables

A rich set of objective variables measuring various domains of cognitive health is collected during the CAPI stage and the nurse-led health assessment in TILDA. Table 1 presents descriptive statistics for the cognitive health variables tested in this study.

**Table 1:** Descriptive statistics, cognitive health outcome variables (TILDA)

Variable	Units	n	Mean ± SD	Min.	Max.
*Global cognitive function:*					
Montreal Cognitive Assessment ^a^	Error score	1407	4.2 ± 3.5	0.0	24.0
Mini Mental State Examination ^b^	Error score	1678	1.4 ± 2.0	0.0	26.0
*Executive function:*					
Animal Naming Test ^b^	Score	1667	19.5 ± 5.8	0.0	44.0
Colour Trail Test 2 ^a^	Time (s)	1394	109.6 ± 46.2	43.5	645.1
*Memory:*					
Immediate recall ^b^	Score	1678	6.1 ± 1.7	0.0	10.0
Delayed recall ^b^	Score	1679	6.3 ± 2.6	0.0	10.0
*Processing speed:*					
Choice Reaction Time ^a^	Total time (ms)	1322	815.5 ± 401.4	474.9	4205.3
Colour Trail Test 1 ^a^	Time (s)	1412	57.5 ± 34.8	0.7	640.5

SD denotes standard deviation.

^a^ Source: TILDA Health Assessment

^b^ Source: TILDA Computer Assisted Personal Interview

#### Mini mental state examination (MMSE)

The MMSE is a screening tool consisting of several exercises designed to measure global cognitive function, and is widely used in the evaluation of dementia. It assesses various cognitive domains: attention and concentration; memory, language, visuospatial ability; calculations; and orientation. Respondents are scored out of 30, with a score of 26 or above indicating normal cognitive function [48]. When studying links between mobility and cognitive function among the TILDA sample of older adults, Donoghue et al. (2018) used the MMSE as the primary measure of global cognition [49]. Another example of the use of the MMSE is in detecting cognitive impairment following acute stroke [50].

#### Montreal cognitive assessment (MoCA)

The MoCA is another global cognitive screening tool assessing eight cognitive functions including: memory; visuospatial ability; executive function; attention; concentration; language; and orientation to time and place. It is utilised to assist physicians in detecting mild cognitive impairment. As in the MMSE, respondents are given a score out of 30, with a score of 26 or above indicating normal cognitive function. The MoCA was developed due to difficulties in detecting mild cognitive impairment using the MMSE, with the MoCA demonstrated to be more sensitive to mild cognitive deficits in older adults. Differences between the two tests include more numerous and demanding tasks to test executive function, language and visuospatial processing in the MoCA [50, 51]. In addition to the MMSE, the Donoghue et al. (2018) study used the MoCA as an alternative measure of global cognition [49].

#### Choice reaction time (CRT)

The CRT test is designed to measure the respondent’s processing speed and concentration. It is a computer-based task, where respondents press a central button until a stimulus appears on screen. On seeing the stimulus, the word ‘yes’ or the word ‘no’, respondents must release the central button and press the target button corresponding to the stimulus before returning to the central button. This is repeated around 100 times, and the mean cognitive response time (the time taken to release the central button) and mean movement time (the time between releasing the central button and pressing the target button) are recorded and combined to give a mean total response time [49]. Donoghue et al. (2018) employ the CRT test as one measure of processing speed among the TILDA sample [49].

#### Animal naming test (ANT)

In the ANT, respondents are given one minute to name as many animals as they can. It is regarded as a test of executive function, as a high score requires good organisation of verbal retrieval and recall. A score of less than 15 named animals is has been viewed as an indication of mild cognitive impairment [52]. The Donoghue et al. (2018) study used the ANT to examine executive function among TILDA respondents [49]. A version of the ANT has also been proposed as a rapid screening test for a decrease in brain function due to liver disease [53].

#### Word recall

TILDA uses both immediate and delayed word recall to measure episodic memory. Respondents are asked to repeat a list of ten words in an interview, and to repeat the same list of words approximately 15 min later [49]. Donoghue et al. (2018) used both recall scores to test episodic memory among older adults [49]. Another example of the use of word recall tests is Coen et al. (1997), a study that determines whether the test can detect mild Alzheimer’s disease [54].

#### Colour trails test (CTT)

In a test of selective attention, mental flexibility, visual spatial skills and motor speed, respondents are timed drawing two trails. In the first test (CTT-1), measuring processing speed, the respondent is timed drawing a line that connects circles numbered one to 25 in consecutive order, without being instructed that the colour of the circles alternates with each succeeding number. In the second test (CTT-2), measuring executive function, respondents are again timed drawing a line between numbered circles in consecutive order, but this time alternating between colours as each number now appears twice. Respondents are notified of any errors and these must be corrected before continuing, and the length of time taken to complete each trail is recorded [55]. The Donoghue et al. (2018) study used CTT-1 and CTT-2 as alternative measures of processing speed and executive function respectively [49]. Another study, which examined the impact of an exercise programme on cognitive function among older adults, also used CTT-2 to measure executive function [56].

### Estimating environmental noise exposure

Using the Predictor-LimA Advanced V2019.02 software package (released May 2019), environmental noise exposure levels were estimated in decibels (dB) by running the ‘Common Noise Assessment Methods in the EU’ (CNOSSOS-EU) methodology, a common framework for strategic noise mapping and population exposure estimation developed for EU Member States [1]. For detailed model specifications, the calculation method is described within EU Directive 2015/996 for road, rail and industrial noise [42] which replaced Annex II of Commission Directive (EU) 2002/49, with further technical information provided by Murphy et al. [43]. Predictor-LimA Advanced V2019.02 is ISO 17534 Quality Assured and certified to perform calculations for CNOSSOS-EU by means of validated results against test cases. According to CNOSSOS-EU and in line with Commission Directive (EU) 2015/996, noise receiver points were assigned to the façades of all buildings in Dublin and Cork. Input data, including source and propagation data as required by CNOSSOS-EU, were obtained from the designated noise mapping bodies for both cities. Source data included reference conditions and traffic count data for each road segment categorised by vehicle type according to the WG-AEN GPG v2 Toolkit [57]. One source centre-line was modelled for each road segment. In the case of carriageways with a central median, a centre line was used for each carriageway. Vehicle speed was set to speed limit values for each road segment. Propagation data included building polygons extruded by height, air temperature, road surface conditions and gradient. These existing digital datasets were collated over several years between 2012 and 2016 and the majority were employed in the most recent round of Strategic Noise Mapping in 2017 [1]. CNOSSOS-EU endeavours to minimise the use of estimated input data in favour of measured inputs. As is the case in all Member States, not all required input data was readily available for input into the CNOSSOS-EU model. Where such data gaps arose, we used the WG-AEN GPG v2 Toolkit and recommendations set out in a data needs assessment funded by the Irish Environmental Protection Agency. This data needs assessment forms part of a wider project on transitioning to Strategic Noise Mapping under CNOSSOS-EU (Noise-Adapt) and deals with data gaps and treatment for CNOSSOS-EU in the Irish case [43]. This analysis thereby employed the most comprehensive datasets available for the cities included in the analysis.

**Fig. 1:**
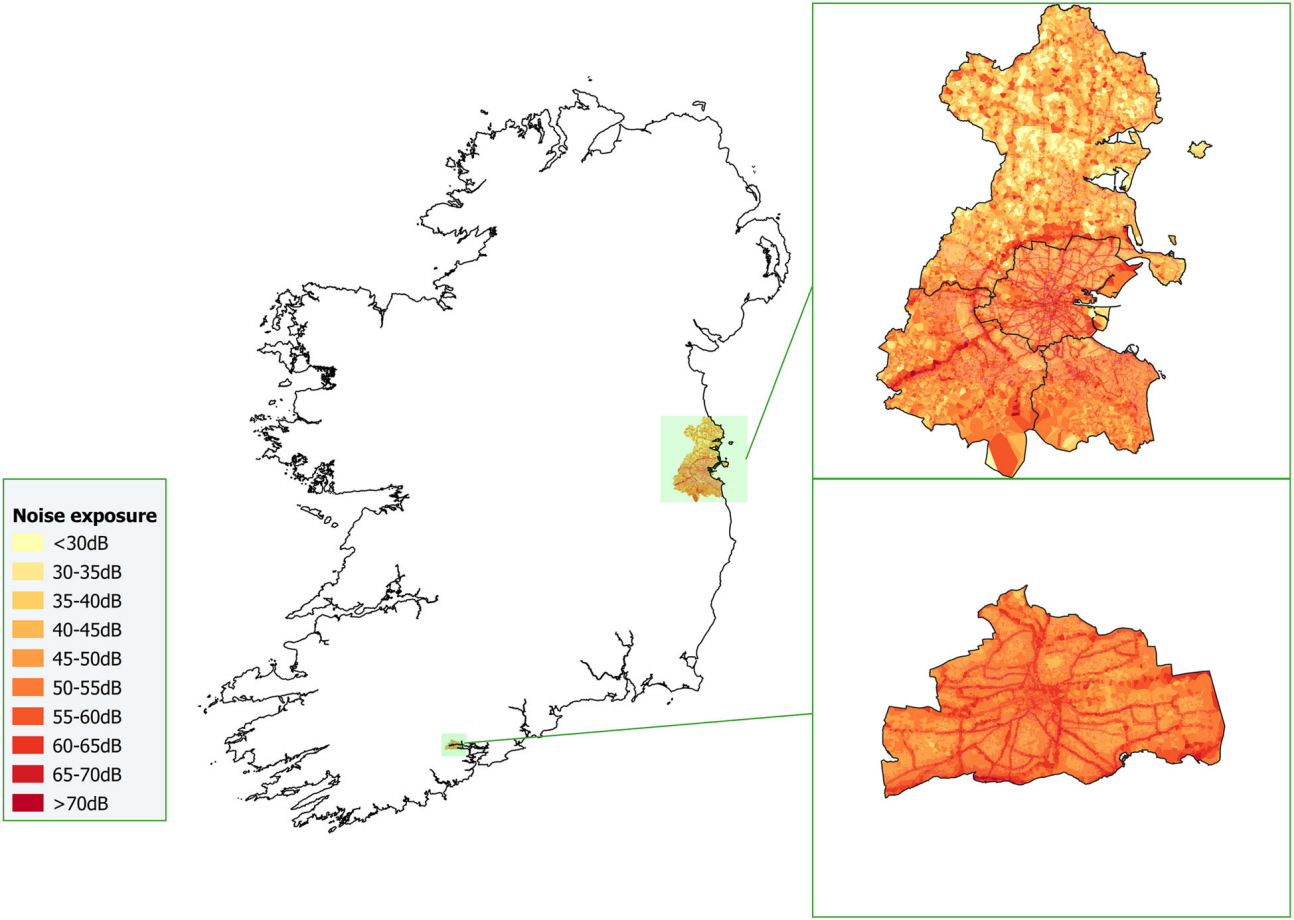
Night-time noise exposure (L_night_) at the most exposed façade of residential buildings, Dublin and Cork

We calculated L_night_ for night-time periods for each receiver point. For L_night_, ‘night-time’ is defined as the eight-hour period between 23:00 and 07:00. While we have no data on the times at which TILDA respondents are usually in their residence, we contend that it is reasonable to assume that the majority of respondents spend this overnight period at home. In so-doing, and in the absence of data on bedroom location or orientation, we followed previous literature in applying night-time noise exposure at the most exposed façade of each building as our household-level exposure variable [5]. Descriptive statistics for our exposure variable, L_night_, are included in Table 3. Based on this methodology, L_night_ exposure values for Dublin and Cork are illustrated in Fig. 1.

### Covariates: socio-demographics and behaviour

To identify any associations between environmental noise and cognition that are independent of potentially confounding sociodemographic or behavioural characteristics, we adjusted our models for these individual-level characteristics using variables collected in TILDA. Table 2 presents descriptive statistics of these variables.

**Table 2:** Descriptive statistics, potentially confounding characteristics (TILDA)

Variable	Category	n (%)
Age class	50–64	707 (41.4)
	65–74	594 (34.8)
	75–84	323 (18.9)
	85+	82 (4.8)
Female		943 (55.3)
Married		1129 (66.2)
Employment	Employed	485 (28.4)
	Retired	921 (54.0)
	Other	300 (17.6)
Highest education ^a^	Primary or none	404 (23.7)
	Secondary	582 (34.1)
	Tertiary or above	720 (42.2)
Household income	€0 to €10,000	55 (3.2)
	€10,000 to €20,000	256 (15.0)
	€20,000 to €40,000	490 (28.7)
	€40,000 to €70,000	373 (21.9)
	€70,000 to €120,000	180 (10.6)
	€120,000 or above	34 (2.0)
	Missing data	318 (18.6)
Physical activity ^b^	None	182 (10.7)
	Low	478 (28.0)
	Moderate	695 (40.7)
	High	351 (20.6)
Social connectedness ^c^	Most isolated	141 (8.3)
	Moderately isolated	501 (29.4)
	Moderately integrated	681 (39.9)
	Most integrated	365 (21.4)
	Missing data	18 (1.1)
Long-term health limitation		431 (25.3)
Alcohol problem ^d^		217 (12.7)
Polypharmacy ^e^		452 (26.5)
Total observations		1706 (100.0)

Source: TILDA

^a^ In Ireland, primary education generally caters for students between 4 and 11 years, and secondary education between 12 and 18 years

^b^ Physical activity is categorised on the International Physical Activity Questionnaire scale [58]

^c^ Social connectedness is categorised on the Berkman-Syme Social Network Index [59]

^d^ Alcohol problem is determined by the respondent’s outcome on the CAGE problematic alcohol scale [60]

^e^ In TILDA, polypharmacy indicates the regular use of at least 5 different medications

We followed the literature in adjusting our models for age, gender, socio-economic status, alcohol consumption and physical activity [13]. We measured socio-economic status using both net household income and the highest level of education attained. Alcohol consumption was included using the outcome of the CAGE problematic alcohol scale [60], and physical activity was captured using the International Physical Activity Questionnaire [58]. We further adjusted for residential density within a one-kilometre radius, for employment status, for general health status as measured by having a long-term health limitation and by the use of five or more medications on a regular basis, and for social connectedness using the Berkman-Syme Social Network Index [59]. Since all members of TILDA sample households who are over the age of 50 are surveyed, it should be noted that with the exception of household income, all other sociodemographic and behavioural covariates are measured at the individual level.

### Covariates: ambient air quality

We also adjusted our models for exposure to air pollution in order to isolate any independent associations between environmental noise and cognition. We included a household-level variable measuring air pollution at each respondent’s residence for this purpose; descriptive statistics are presented in Table 3. The correlation between night-time road traffic noise exposure and our measure of air pollution is 0.69.

**Table 3:** Descriptive statistics, pollution exposure variables

Variable of exposure	Units	n	Mean ± SD	Min.	Max.
Night-time noise (L_night_)	dB (A)	1706	51.4 ± 5.1	35.7	69.3
NO_2_^a^	μg m^−3^	728	15.6 ± 3.9	8.0	38.6

SD denotes standard deviation.

^a^ Source: Environmental Protection Agency, Ireland

The Irish Environmental Protection Agency assessed urban air quality in Dublin city as a pilot exercise within the Forum for Air Quality Modelling in Europe (FAIRMODE), a recently developed European methodological approach to air quality measurement [61]. Ground-level nitrogen dioxide (NO_2_) exposure was modelled in micrograms per cubic metre air (μg m^− 3^) for 2015, exploiting land, meteorological, and traffic and industrial emissions characteristics. This exercise created a high-resolution map of exposure that can be linked to other geo-coded datasets such as TILDA. Further details on this data are presented by Aves and Williams [61].

We employed this measure of NO_2_ as a proxy for exposure to air pollution more broadly. Data on ambient air pollution was only available to the study for a sub-sample of 728 respondents in Dublin city. We were thus limited to adjusting for air pollution in a sub-sample analysis. This sub-sample cannot be assumed to be representative of our full sample, and this is a limitation of this study.

### Statistical approach

For each measure of cognitive function, we ran a regression model with estimated road traffic noise exposure as an independent variable. The sample size of each model was restricted to respondents for whom data was available on the respective cognitive outcome. Noise exposure and exposure to air pollution were categorised using quintiles as a condition of linking geographic data to TILDA to protect anonymity (the use of quintiles ensured that an equal number of respondents were in each of the five categories of exposure), and to allow for potential non-linear effects in the relationship between exposure and cognitive function.

We used ordinary least squares linear regression models for all cognitive tests, with standard errors clustered at the household level. Choice of model specification was based on a comparison of values for the Akaike Information Criterion and Bayesian Information Criterion for Gaussian, Poisson and negative binomial identities for each outcome variable. We followed Donoghue et al. [49] in calculating the number of errors made in each the MMSE and the MoCA for each participant as the outcome variables for these tests due to potential ceiling effects when using the score itself. This was achieved by subtracting each participant’s score from the maximum score, 30. Given the different units of measurement in the various cognitive measures, outcome variables were standardised using z-scores.

These models were adjusted for individual sociodemographic and behavioural characteristics, allowing us to identify any relationships that are independent of differences in these characteristics. This quantitative technique tested the hypothesis that environmental noise is associated with cognitive health. Our residential density and household income variables were included in logarithm form to better account for their potential relationship with cognitive function. In cases where data was missing on socio-demographic or behavioural covariates for some observations, a “missing” category was added and these observations were included in regression models (see Table 2). For each model, a backward selection process using F-tests was employed to remove covariates that did not impact the model.

We report our results as average marginal effects for quintiles of noise exposure relative to the lowest quintile of exposure, the reference category. The average marginal effect associated with a quintile of noise exposure is the average of predicted changes in the outcome variable when moving from the lowest quintile of noise exposure, holding all other covariates constant. As outcome variables were standardised using z-scores, reported average marginal effects can be interpreted as proportions of a standard deviation. *P*-values, indicating the probability of obtaining results as extreme as the observed results under the null hypothesis, are also reported.

When considering statistical significance, a *p*-value lower than 0.05 is typically regarded as indicative of statistical significance at the 95% confidence level. As several different cognitive health outcomes are tested for associations with noise exposure using the same sample, however, the probability of a false positive (Type 1 error) is elevated. We estimated using the Bonferroni method that this 0.05 threshold is lowered to 0.002 for statistical significance at the 95% level [62].

### Robustness checks

In addition to using night-time noise exposure (L_night_), we ran regression models of our cognitive health outcomes using 24-h noise exposure (L_den_) to check the sensitivity of any statistically significant results to the choice of noise exposure variable. As we measured noise exposure at the most exposed façade for this statistical analysis, we also ran a regression model for any outcome found to be associated with noise including night-time noise exposure (L_night_) at the least exposed façade of residences.

An alternative way of examining the association between noise exposure and performance in the ANT is to consider the probability of a respondent achieving a score indicative of mild cognitive impairment (less than 15) using a logistic regression model. We ran a logistic regression model of this probability to check the robustness of our main result in relation to the ANT.

At the outset of this analysis, we excluded 12 respondents exposed to either railway or aircraft noise at night in order to isolate any associations specifically with road traffic noise. As a further robustness check, we also ran our models including these respondents. For each cognitive outcome, a univariate regression model was also run including noise exposure as the only independent variable.

Finally, focusing on a sub-sample of 728 respondents who live in Dublin city allowed us to further adjust our model for exposure to ambient air quality, using NO_2_ as a proxy for general air pollution. Model specifications that included each PM_2.5_ and PM_10_ as alternative measures of air pollution exposure instead of NO_2_ were also ran using this sub-sample.

## Results

The results of our primary analysis, fully adjusted for socio-demographic and behavioural characteristics, are summarised for our noise exposure variable in Table 4, in the form of average marginal effects for quintiles of noise exposure relative to the lowest quintile of exposure, the reference category. A full set of results is presented for the ANT model in Table B1 (Specification I) of Appendix B (see Additional File 1).

**Table 4:** Average marginal effects of noise exposure (L_night_)

		*dy/dx* (95% C.I.)	p-value	*dy/dx* (95% C.I.)	p-value
		MMSE errors		MoCA errors	
Noise	35.7–45.8 dB(A)	[ref.]		[ref.]	
	45.9–48.9 dB(A)	0.012 (− 0.109, 0.133)	0.847	− 0.151 (− 0.290, − 0.013)	0.034
	49.0–51.2 dB(A)	0.198 (0.066, 0.329)	0.003	−0.011 (− 0.153, 0.130)	0.875
	51.3–53.7 dB(A)	−0.030 (− 0.150, 0.091)	0.628	− 0.123 (− 0.261, 0.014)	0.078
	53.8–69.3 dB(A)	0.108 (− 0.030, 0.247)	0.125	− 0.011 (− 0.160, 0.138)	0.886
N		1678		1407	
		ANT score		CTT-2 time	
Noise	35.7–45.8 dB(A)	[ref.]		[ref.]	
	45.9–48.9 dB(A)	−0.053 (−0.195, 0.088)	0.457	−0.099 (− 0.233, 0.035)	0.147
	49.0–51.2 dB(A)	− 0.117 (− 0.259, 0.025)	0.107	− 0.083 (− 0.229, 0.064)	0.269
	51.3–53.7 dB(A)	− 0.115 (− 0.250, 0.020)	0.096	0.037 (− 0.122, 0.196)	0.648
	53.8–69.3 dB(A)	− 0.209 (− 0.346, − 0.072)	0.003	0.059 (− 0.078, 0.195)	0.399
N		1667		1394	
		Immediate recall score		Delayed recall score	
Noise	35.7–45.8 dB(A)	[ref.]		[ref.]	
	45.9–48.9 dB(A)	0.074 (−0.062, 0.211)	0.284	0.137 (0.005, 0.270)	0.043
	49.0–51.2 dB(A)	0.031 (−0.103, 0.164)	0.654	0.055 (− 0.085, 0.195)	0.442
	51.3–53.7 dB(A)	0.110 (−0.026, 0.246)	0.114	0.087 (− 0.048, 0.223)	0.207
	53.8–69.3 dB(A)	0.041 (− 0.099, 0.180)	0.570	0.054 (− 0.079, 0.187)	0.424
N		1678		1679	
		CTT-1 time		CRT total time	
Noise	35.7–45.8 dB(A)	[ref.]		[ref.]	
	45.9–48.9 dB(A)	−0.092 (−0.208, 0.025)	0.123	0.000 (−0.149, 0.149)	1.000
	49.0–51.2 dB(A)	0.078 (−0.110, 0.266)	0.413	0.035 (−0.117, 0.186)	0.653
	51.3–53.7 dB(A)	−0.028 (− 0.151, 0.094)	0.649	0.083 (− 0.080, 0.245)	0.319
	53.8–69.3 dB(A)	−0.030 (− 0.148, 0.088)	0.616	0.087 (− 0.082, 0.255)	0.315
N		1412		1322	

Noise exposure is categorised using quintiles. Cognitive health outcome variables standardised using z-scores. Results correspond to models that adjust for socio-demographic, behavioural and health characteristics. C.I. denotes confidence interval

Table 4 reveals a mixed picture. From an initial examination of global cognitive function using the MMSE, there appears to be little evidence of a relationship with noise exposure. Respondents in the third quintile of noise exposure make more errors in the MMSE than respondents in the lowest quintile. This result suggests that moving from the lowest quintile of noise exposure to the middle quintile is associated with an increase in the number of errors made in the MMSE, to the magnitude of 0.198 of a standard deviation. This association is not reflected in other quintiles of noise exposure, and this represents an unclear result.

We exploited the rich data on specific domains of cognitive function captured by TILDA to investigate associations between road traffic noise and more specific domains of cognitive function, specifically executive function, memory, and processing speed. Using the ANT to assess executive function, we find some evidence of a negative relationship between noise exposure and cognitive performance, with an average marginal effect at the highest quintile that is supported by a low *p*-value (although not statistically significant at the 95% level when accounting for multiple testing). The average marginal effect shows a reduced score of 0.209 of a standard deviation for respondents in the highest quintile. This suggests a negative association between noise exposure and executive function: respondents with the highest levels of noise exposure achieved lower ANT scores than respondents with the lowest levels of exposure. The CTT-2 also tests executive function, and there is no evidence of an association between noise and time achieved in the CTT-2, with no systematic differences in scores between respondents with different levels of noise exposure. This may be due to a smaller sample size for the CTT-2 relative to the ANT, or due to it being a different test of executive function.

In the case of memory, using the immediate and delayed recall tests, we cannot reject the null hypothesis of there being no association with noise exposure, with no differences found in scores between respondents based on differences in noise exposure. Similarly, assessing processing speed using the CTT-1 and the total time in the CRT, we find no evidence of an association with noise exposure.

The results indicating a possible negative association between noise exposure and performance in the ANT require further scrutiny. Table B1 (Specification II) in Appendix B (see Additional File 1) presents results for a regression model of the ANT score that included 24-h noise exposure (L_den_) at the most exposed façade as the noise exposure variable, instead of night-time exposure (L_night_). This shows broadly similar results to our main ANT model, with a reduced ANT score associated with the highest quintile of 24-h noise exposure relative to the lowest quintile, indicating that the result is not sensitive to the choice of noise exposure variable at the most exposed façade. A regression model of the ANT score that included night-time noise exposure at the least exposed façade, however, showed no association between noise and executive function.

In a logistic regression model of the probability of a respondent achieving an ANT score indicative of mild cognitive impairment (lower than 15), we found no evidence of a relationship with noise exposure, with respondents with higher levels of noise exposure no more likely to achieve a score lower than 15 than other respondents. This may suggest that the possible relationship between noise exposure and performance in the ANT suggested by our main results is not sufficiently pronounced to manifest itself in an association with the probability of achieving a score indicative of mild cognitive impairment.

We confirm that our results are unchanged when 12 respondents exposed to either railway or aircraft noise at night are included in the models. An association between noise exposure and performance in the ANT score was also evident in a univariate model including noise exposure as the only independent variable.

Results for the sub-sample analysis of the ANT for 728 respondents who live in Dublin city, which additionally includes exposure to air pollution in the model, are presented in Table B2 in Appendix B (see Additional File 1). In this sub-sample analysis, there is little evidence of an association between noise exposure and performance in the ANT. An association between ANT performance and exposure to NO_2_ pollution is evident, with a reduction of 0.472 of a standard deviation in the ANT score associated with being in the highest quintile of noise exposure relative to the lowest. This association with air pollution is not reflected in models using PM_2.5_ or PM_10_ instead of NO_2_, however.

## Discussion

Environmental noise is an inevitable feature of daily living and is particularly relevant in urban areas. Given increasing urbanisation globally, awareness of the potential impact of environmental noise on public health is crucial [1]. This paper contributes to a growing evidence base in relation to road traffic noise and cognitive health among older adults and can thus help to broaden our understanding of this issue.

Overall, the results of this study indicate that long term exposure to road traffic noise may be negatively associated with executive function as measured by the ANT among our sample of older adults. The magnitude of this relationship is considerable, with a reduced ANT score of 0.209 of a standard deviation associated with respondents in the highest quintile of noise exposure relative to those in the lowest quintile, only slightly smaller than the average marginal effect of having completed secondary education relative to having only primary education. This association between road traffic noise and executive function is independent of various individual socio-demographic and behavioural characteristics. However, when accounting for multiple testing, the association is not statistically significant at the 95% level. Residential proximity to a major roadway was associated with poorer performance in executive function in a previous study [22]. The sensitivity of our result to the measurement of noise exposure at the most exposed façade of a respondent’s residence instead of the least exposed façade could indicate that a possible association between noise and the ANT score is mitigated to some extent between the most exposed and the least exposed façade.

An unclear signal for noise exposure is detected from a model with the measure of global cognitive function as the dependent variable, the MMSE, although this is not consistent across different quintiles of noise exposure and is not reflected in a model of the MoCA, and thus may be spurious. In the literature, global measures of cognition similar to the MMSE and the MoCA were found to be associated with long term exposure to road traffic noise [13] or residential proximity to a major roadway [22]. Overall, however, in our study of older adults the results for these two tests of global cognitive function are inconclusive. This inconclusive finding for global executive function could be considered consistent with our results of models examining specific domains of cognition, in that some evidence of a negative relationship is found with executive function but no evidence is found for a relationship with memory or with processing speed. Residential proximity to a major roadway was associated with verbal memory in the literature [22], although Wellenius et al.’s measure of exposure encompassed other environmental stressors in addition to road traffic noise. Road traffic noise has previously been associated with impaired recall in children [27, 63], whereas our study focuses on older adults.

When adjusting for exposure to air pollution in a sub-sample analysis, however, exposure to road traffic noise does not appear to be associated with performance in the ANT, with some evidence of an association found instead between ANT performance and air pollution exposure. In our main ANT model, where air pollution is omitted, road traffic noise may actually be detecting an association with air pollution. The correlation between the noise pollution and air pollution variables is relatively strong at 0.69 among the sub-sample for which air pollution data is available. Alternatively, it may be that cognitive function is in fact associated with proximity to traffic, and that our measure of air pollution is a more refined measure of this than our measure of road traffic noise. However, while it additionally adjusts our model for exposure to air pollution, this sub-sample analysis is ultimately not comparable with our main models as the sample size is considerably reduced, and moreover relies on a sub-sample that is not representative of our full sample. Therefore, it is not possible to draw any definitive conclusions from this sub-sample analysis.

This paper suffers from some limitations. First, the specificity of this research may also be considered a limitation, in that while we contribute to the evidence base in relation to older adults, our results cannot be generalised to the whole population. Further research is required to extend the understanding of associations between road traffic noise exposure and cognitive health for other demographic groups. In addition, while TILDA allowed us to explore associations between noise exposure and a wide range of measures of cognitive function, we could not empirically study the mechanisms behind any association. An empirical examination of the potential mechanisms underlying a possible association between exposure to environmental noise and executive function may thus be an avenue for further research.

The noise exposure modelling resulted in a measure that represents average night-time exposure at the most exposed façade of the respondent’s residence, L_night_. In the absence of information on the layout of respondents’ homes or their activity patterns, we also necessarily assume that night-time exposure at the most exposed façade is a good proxy for an individual’s exposure to noise. A further limitation is that this study’s sample size may not have afforded sufficient statistical power to detect noise-cognition associations, particularly in the sub-sample analysis that adjusted for air pollution exposure. This issue is particularly evident when accounting for multiple testing in the statistical analysis.

It is also the case that due to limitations in data availability, data on cognitive health outcomes, as well as socio-demographic and behavioural covariates, were collected by TILDA during 2014 and 2015, while data on road traffic noise exposure was modelled based on input data collated between 2012 and 2016. This limitation would only be expected to affect results if the noise modelling input data changed significantly during this period, however, and this is considered unlikely. It should also be noted that the response rate in TILDA varied by survey question or cognitive test. In particular, in this study, data on household income was missing for 18.6% of our sample (see Table 2). While other covariates, such as employment status and education, were also included in regression models to adjust for socio-economic status, this incomplete data on income is a limitation of the study. However, that the association between noise exposure and ANT performance persisted in a univariate model (thus omitting all other covariates) indicates that this issue did not substantially affect the study’s findings.

This study was cross sectional in nature and thus cannot make any causal inference. Subject to data availability, future research could exploit longitudinal data to move closer to causality. Finally, due to data limitations, we were only able to adjust models for air pollution for a sub-sample in Dublin city that was not representative of our full sample of TILDA respondents from Dublin and Cork. Subject to data availability, applying our model of executive function including both noise and air pollution to a larger sample would be beneficial.

## Conclusions

We found some evidence that road traffic noise exposure was negatively associated with executive function among our sample of older adults as measured by the ANT, with respondents with the highest levels of noise exposure achieving lower scores. This association appeared to be accounted for by exposure to air pollution when focusing on an unrepresentative sub-sample, however. We did not find evidence of an association between noise exposure and either global cognition, memory or processing speed.

This paper makes several valuable contributions to the literature. First, by spatially linking high quality modelled noise pollution data based on the new CNOSSOS-EU standard to TILDA, we exploited a rich dataset that included measures of various domains of cognition in addition to detailed socio-demographic and behavioural characteristics. This enabled us to identify associations between road traffic noise and cognition that are independent of these characteristics. Second, this research was focused on a sample of older adults, considered to be a group particularly vulnerable to environmental stressors though not yet extensively studied in the literature. Furthermore, we included data on air pollution in a sub-sample analysis in an attempt to isolate any associations that are independent of our measure of this other environmental stressor.

## Supplementary Information

**Additional File 1.**

## Data Availability

Researchers interested in using the TILDA data used in this paper may access the data on request via www.tilda.ie. Statistical analysis was conducted using the statistical software programme, Stata 14. Code for this analysis is available on request from the authors.

## Abbreviations

ANT: Animal Naming Test
CAPI: Computer Assisted Personal Interviewing
CERAD-Plus: Consortium to Establish a Registry on Alzheimer’s Disease
CES-D: Center for Epidemiologic Studies Depression scale
CI: Confidence interval
CNOSSOS-EU: Common Noise Assessment Methods in the EU
CTT: Colour Trails Test
CRT: Choice Reaction Time
DALYs: Disability-Adjusted Life Years
dB: Decibels
EPA: Environmental Protection Agency
GIS: Geographic Information System
HRS: Health and Retirement Survey
L_night_: Night-time noise level
MMSE: Mini Mental State Examination
MoCA: Montreal Cognitive Assessment
NO_2_: Nitrogen dioxide
TILDA: The Irish Longitudinal Study on Ageing
WHO: World Health Organization
μg m^− 3^: Micrograms per cubic metre air

## References

[1] Murphy E, Faulkner JP, Douglas O. Current State-of-the-Art and New Directions in Strategic Environmental Noise Mapping. Curr Pollut Rep [Internet]. 2020 [cited 2020 Apr 17]; Available from: http://link.springer.com/10.1007/s40726-020-00141-9

[2] WHO. Environmental noise guidelines for the European Region [Internet]. 2018 [cited 2020 Mar 2]. Available from: http://www.euro.who.int/__data/assets/pdf_file/0008/383921/noise-guidelines-eng.pdf?ua=1

[3] van Kempen E, Casas M, Pershagen G, Foraster M (2018). WHO environmental noise guidelines for the European region: a systematic review on environmental noise and cardiovascular and metabolic effects: a summary. Int J Environ Res Public Health.

[4] Guski R, Schreckenberg D, Schuemer R (2017). WHO environmental noise guidelines for the European region: a systematic review on environmental noise and annoyance. Int J Environ Res Public Health.

[5] Basner M, McGuire S (2018). WHO environmental noise guidelines for the European region: a systematic review on environmental noise and effects on sleep. Int J Environ Res Public Health.

[6] Clark C, Paunovic K (2018). WHO environmental noise guidelines for the European region: a systematic review on environmental noise and cognition. Int J Environ Res Public Health.

[7] Śliwińska-Kowalska M, Zaborowski K (2017). WHO environmental noise guidelines for the European region: a systematic review on environmental noise and permanent hearing loss and tinnitus. Int J Environ Res Public Health.

[8] Nieuwenhuijsen M, Ristovska G, Dadvand P (2017). WHO environmental noise guidelines for the European region: a systematic review on environmental noise and adverse birth outcomes. Int J Environ Res Public Health.

[9] Clark C, Paunovic K (2018). WHO environmental noise guidelines for the European region: a systematic review on environmental noise and quality of life, wellbeing and mental health. Int J Environ Res Public Health.

[10] European Environment Agency. Environmental noise in Europe, 2020 [Internet]. Luxembourg: Publications Office; 2020 [cited 2021 ]. Available from: https://data.europa.eu/doi/10.2800/686249

[11] Douglas O, Murphy E (2020). Assessing the treatment of potential effect modifiers informing World Health Organisation guidelines for environmental noise. Int J Environ Res Public Health.

[12] van Kamp I, Davies H (2013). Noise and health in vulnerable groups: a review. Noise Health.

[13] Tzivian L, Jokisch M, Winkler A, Weimar C, Hennig F, Sugiri D, Soppa VJ, Dragano N, Erbel R, Jöckel KH, Moebus S, Hoffmann B, Heinz Nixdorf Recall Study Group (2017). Associations of long-term exposure to air pollution and road traffic noise with cognitive function—an analysis of effect measure modification. Environ Int.

[14] Tzivian L, Winkler A, Dlugaj M, Schikowski T, Vossoughi M, Fuks K, Weinmayr G, Hoffmann B (2015). Effect of long-term outdoor air pollution and noise on cognitive and psychological functions in adults. Int J Hyg Environ Health.

[15] Clark C, Crumpler C, Notley H (2020). Evidence for environmental noise effects on health for the United Kingdom policy context: a systematic review of the effects of environmental noise on mental health, wellbeing, quality of life, Cancer, dementia, birth, reproductive outcomes, and cognition. Int J Environ Res Public Health.

[16] Yu Y, Paul K, Arah OA, Mayeda ER, Wu J, Lee E, Shih IF, Su J, Jerrett M, Haan M, Ritz B (2020). Air pollution, noise exposure, and metabolic syndrome – a cohort study in elderly Mexican-Americans in Sacramento area. Environ Int.

[17] Fuks KB, Wigmann C, Altug H, Schikowski T (2019). Road traffic noise at the residence, annoyance, and cognitive function in elderly women. Int J Environ Res Public Health.

[18] Tzivian L, Dlugaj M, Winkler A, Weinmayr G, Hennig F, Fuks KB, Vossoughi M, Schikowski T, Weimar C, Erbel R, Jöckel KH, Moebus S, Hoffmann B, on behalf of the Heinz Nixdorf Recall study Investigative Group (2016). Long-term air pollution and traffic noise exposures and mild cognitive impairment in older adults: a cross-sectional analysis of the Heinz Nixdorf recall study. Environ Health Perspect.

[19] Kulick ER, Wellenius GA, Boehme AK, Joyce NR, Schupf N, Kaufman JD, Mayeux R, Sacco RL, Manly JJ, Elkind MSV (2020). Long-term exposure to air pollution and trajectories of cognitive decline among older adults. Neurology..

[20] Tonne C, Elbaz A, Beevers S, Singh-Manoux A. Traffic-related Air Pollution in Relation to Cognitive Function in Older Adults: Epidemiology 2014;25(5):674–681, DOI: 10.1097/EDE.0000000000000144. 10.1097/EDE.0000000000000144 PMC4162337 25036434

[21] Carey IM, Anderson HR, Atkinson RW, Beevers SD, Cook DG, Strachan DP, Dajnak D, Gulliver J, Kelly FJ (2018). Are noise and air pollution related to the incidence of dementia? A cohort study in London. England BMJ Open.

[22] Wellenius GA, Boyle LD, Coull BA, Milberg WP, Gryparis A, Schwartz J, Mittleman MA, Lipsitz LA (2012). Residential proximity to nearest major roadway and cognitive function in community-dwelling seniors: results from the MOBILIZE Boston study. J Am Geriatr Soc.

[23] Chen H, Kwong JC, Copes R, Tu K, Villeneuve PJ, van Donkelaar A, Hystad P, Martin RV, Murray BJ, Jessiman B, Wilton AS, Kopp A, Burnett RT (2017). Living near major roads and the incidence of dementia, Parkinson’s disease, and multiple sclerosis: a population-based cohort study. Lancet.

[24] Díaz J, Martínez-Martín P, Rodríguez-Blázquez C, Vázquez B, Forjaz MJ, Ortiz C, Carmona R, Linares C (2018). Short-term association between road traffic noise and healthcare demand generated by Parkinson\textquotesingles disease in Madrid. Spain Gac Sanit.

[25] Tassi P, Rohmer O, Bonnefond A, Margiocchi F, Poisson F, Schimchowitsch S (2013). Long term exposure to nocturnal railway noise produces chronic signs of cognitive deficits and diurnal sleepiness. J Environ Psychol.

[26] Hygge S, Evans GW, Bullinger M (2002). A prospective study of some effects of aircraft noise on cognitive performance in schoolchildren. Psychol Sci.

[27] Stansfeld SA, Berglund B, Clark C, Lopez-Barrio I, Fischer P, Öhrström E, Haines MM, Head J, Hygge S, van Kamp I, Berry BF (2005). Aircraft and road traffic noise and children’s cognition and health: a cross-national study. Lancet.

[28] Brocolini L, Parizet E, Chevret P (2016). Effect of masking noise on cognitive performance and annoyance in open plan offices. Appl Acoust.

[29] Golmohammadi R, Darvishi E, Faradmal J, Poorolajal J, Aliabadi M (2020). Attention and short-term memory during occupational noise exposure considering task difficulty. Appl Acoust.

[30] Czéh B, Müller-Keuker JIH, Rygula R, Abumaria N, Hiemke C, Domenici E, Fuchs E (2007). Chronic social stress inhibits cell proliferation in the adult medial prefrontal cortex: hemispheric asymmetry and reversal by fluoxetine treatment. Neuropsychopharmacology..

[31] Jafari Z, Kolb BE, Mohajerani MH (2018). Chronic traffic noise stress accelerates brain impairment and cognitive decline in mice. Exp Neurol.

[32] Arnsten AFT (2009). Stress signalling pathways that impair prefrontal cortex structure and function. Nat Rev Neurosci.

[33] Jafari Z, Kolb BE, Mohajerani MH (2019). Noise exposure accelerates the risk of cognitive impairment and Alzheimer’s disease: adulthood, gestational, and prenatal mechanistic evidence from animal studies. Neurosci Biobehav Rev.

[34] Schmidt FP, Basner M, Kroger G, Weck S, Schnorbus B, Muttray A, Sariyar M, Binder H, Gori T, Warnholtz A, Munzel T (2013). Effect of nighttime aircraft noise exposure on endothelial function and stress hormone release in healthy adults. Eur Heart J.

[35] Cui B, Gai Z, She X, Wang R, Xi Z (2016). Effects of chronic noise on glucose metabolism and gut microbiota–host inflammatory homeostasis in rats. Sci Rep.

[36] Björntorp P, Rosmond R (2000). Obesity and cortisol. Nutrition..

[37] Passchier-Vermeer W, Passchier WF (2000). Noise exposure and public health. Environ Health Perspect.

[38] Cappuccio FP, D’Elia L, Strazzullo P, Miller MA (2010). Quantity and quality of sleep and incidence of type 2 diabetes: a systematic review and meta-analysis. Diabetes Care.

[39] Chaput J-P, Després J-P, Bouchard C, Tremblay A (2007). Short sleep duration is associated with reduced leptin levels and increased adiposity: results from the Québec family study*. Obesity..

[40] Van Cauter E, Spiegel K, Tasali E, Leproult R (2008). Metabolic consequences of sleep and sleep loss. Sleep Med.

[41] Guo L, Li P, Li H, Colicino E, Colicino S, Wen Y, Zhang R, Feng X, Barrow TM, Cayir A, Baccarelli AA, Byun HM (2017). Effects of environmental noise exposure on DNA methylation in the brain and metabolic health. Environ Res.

[42] European Commission. Directive 2015/996 of 19 May 2015 establishing common noise assessment methods according to Directive 2002/49/EC of the European Parliament and of the Council. [Internet]. 2015. Available from: https://eur-lex.europa.eu/legal-content/EN/TXT/PDF/?uri=CELEX:32015L0996&from=PT

[43] Murphy E, Faulkner JP, Rice HJ, Kennedy J. Transitioning to strategic noise mapping under CNOSSOS-EU (noise-adapt) [internet]. Wexford: Environmental Protection Agency; 2021 [cited 2021 Aug 12]. Available from: https://www.epa.ie/publications/research/epa-research-2030-reports/Research_Report_382.pdf

[44] Donoghue OA, McGarrigle CA, Foley M, Fagan A, Meaney J, Kenny RA (2018). Cohort Profile Update: The Irish Longitudinal Study on Ageing (TILDA). Int J Epidemiol.

[45] Kearney PM, Cronin H, O’Regan C, Kamiya Y, Savva GM, Whelan B (2011). Cohort profile: the Irish longitudinal study on ageing. Int J Epidemiol.

[46] Environmental Protection Agency. Noise Round 3 Rail (Lnight) [Internet]. 2018. Available from: http://www.isde.ie/geonetwork/srv/api/records/72f5b183-40ea-4b68-aa55-0d7ea87a4322/formatters/xsl-view?root=div&output=pdf

[47] Environmental Protection Agency. Noise Round 3 Airport (Lnight) [Internet]. 2018. Available from: http://www.isde.ie/geonetwork/srv/api/records/c2a992d0-e492-424d-8546-4fe765bc272c/formatters/xsl-view?root=div&output=pdf

[48] Folstein MF, Folstein SE, McHugh PR (1975). Mini-mental state. J Psychiatr Res.

[49] Donoghue OA, Feeney J, O’Leary N, Kenny RA (2018). Baseline mobility is not associated with decline in cognitive function in healthy community-dwelling older adults: findings from the Irish longitudinal study on ageing (TILDA). Am J Geriatr Psychiatry.

[50] Dong Y, Sharma VK, Chan BP-L, Venketasubramanian N, Teoh HL, Seet RCS, Tanicala S, Chan YH, Chen C (2010). The Montreal cognitive assessment (MoCA) is superior to the Mini-mental state examination (MMSE) for the detection of vascular cognitive impairment after acute stroke. J Neurol Sci.

[51] Nasreddine ZS, Phillips NA, BÃ©dirian V, Charbonneau S, Whitehead V, Collin I (2005). the Montreal cognitive assessment, MoCA: a brief screening tool for mild cognitive impairment. J Am Geriatr Soc.

[52] Campagna F, Montagnese S, Ridola L, Senzolo M, Schiff S, Rui MD (2017). The animal naming test: an easy tool for the assessment of hepatic encephalopathy. Hepatology..

[53] Labenz C, Beul L, Toenges G, Schattenberg JM, Nagel M, Sprinzl MF, Nguyen-Tat M, Zimmermann T, Huber Y, Marquardt JU, Galle PR, Wörns MA (2019). Validation of the simplified animal naming test as primary screening tool for the diagnosis of covert hepatic encephalopathy. Eur J Intern Med.

[54] Coen RF, Kirby M, Swanwick GRJ, Maguire CP, Walsh JB, Coakley D, O'Neill D, Lawlor BA (1997). Distinguishing between patients with depression or very mild Alzheimer’s disease using the delayed-word-recall test. Dement Geriatr Cogn Disord.

[55] Elkin-Frankston S, Lebowitz B, Kapust L, Hollis A, O’Connor M (2007). The use of the color trails test in the assessment of driver competence: preliminary report of a culture-fair instrument. Arch Clin Neuropsychol.

[56] Anderson-Hanley C, Nimon JP, Westen SC (2010). Cognitive health benefits of strengthening exercise for community-dwelling older adults. J Clin Exp Neuropsychol.

[57] European Commission (2007). Good practice guide for strategic noise mapping and the production of associated data on noise exposure: European Commission working group assessment of exposure to noise (WG-AEN) [internet]. European Commission.

[58] Craig CL, Marshall AL, Sjöström M, Bauman AE, Booth ML, Ainsworth BE (2003). International Physical Activity Questionnaire: 12-Country Reliability and Validity. Med Sci Sports Exerc.

[59] Berkman LF, Syme SL (1979). Social networks, host resistance, and mortality: a nine-year follow-up study of Alameda County residents. Am J Epidemiol.

[60] Ewing JA (1984). Detecting alcoholism: the CAGE questionnaire. JAMA..

[61] Aves C, Williams M (2019). Urban air quality modelling of Dublin [internet]. Cambridge environmental research consultants.

[62] Newson R (2003). The ALSPAC study team. Multiple-test procedures and smile plots. Stata J Promot Commun Stat Stata.

[63] Hygge S (2003). Noise exposure and cognitive performance – children and the elderly as possible risk groups.

