# Supplementary material for: Road traffic noise and cognitive function in older adults: a cross-sectional investigation of The Irish Longitudinal Study on Ageing
Source: BMC Public Health. 2021 Oct 8;21:1814. doi: 10.1186/s12889-021-11853-y (PMC8501545; doi:10.1186/s12889-021-11853-y)
Supplement: Supplementary file 1 — Additional File 1. [file 12889_2021_11853_MOESM1_ESM.pdf]

Appendix A: Sample size

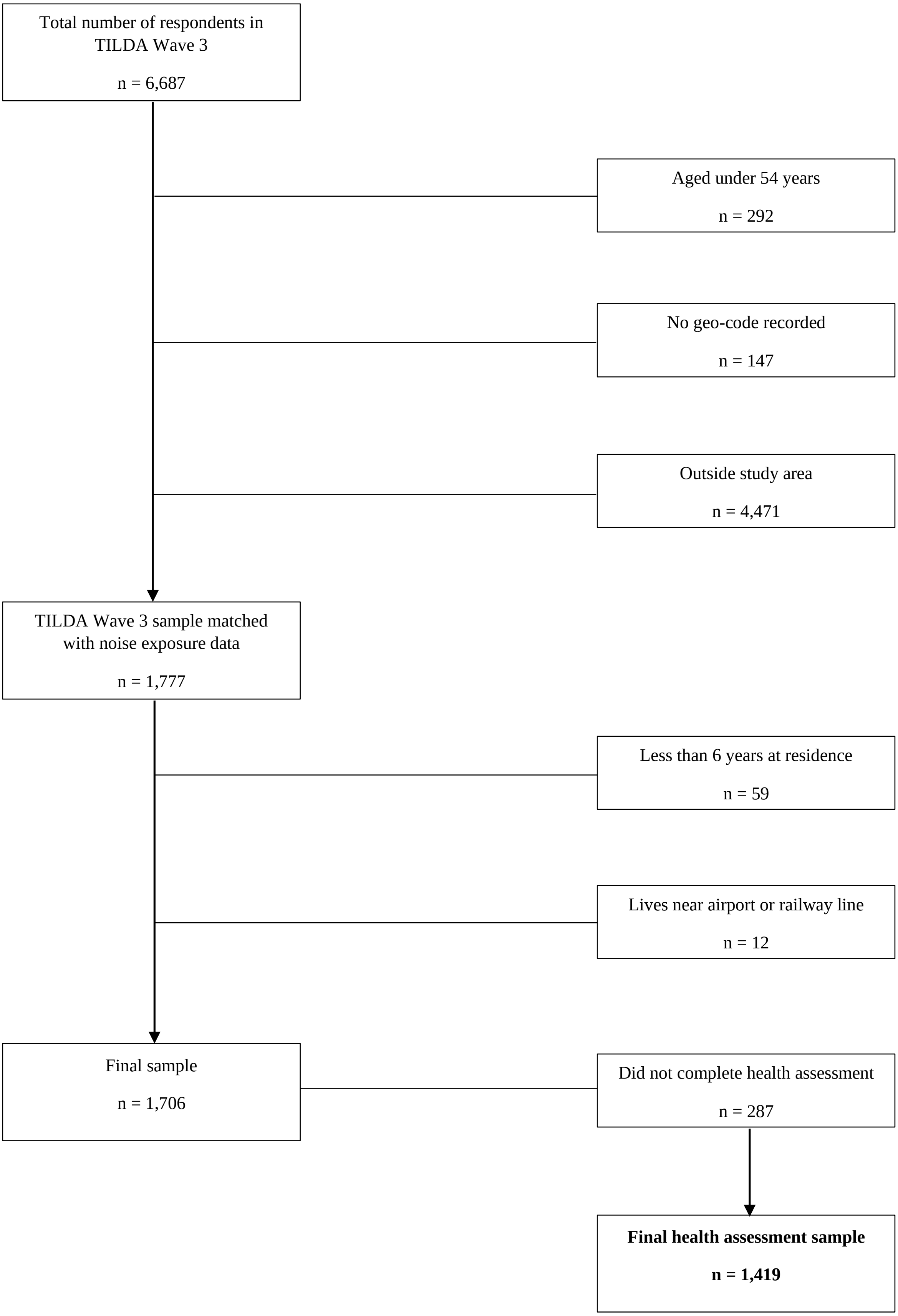

Appendix B: Additional tables

Table B1: Average marginal effects of noise exposure and covariates, ANT score

		(I) ANT score		(II) ANT score	
		dy/dx (95% C.I.)	p-value	*dy/dx* (95% C.I.)	p-value
Noise (L_{night})	Lowest quintile	[ref.]			
	Second quintile	-0.053 (-0.195, 0.088)	0.457		
	Third quintile	-0.117 (-0.259, 0.025)	0.107		
	Fourth quintile	-0.115 (-0.250, 0.020)	0.096		
	Highest quintile	-0.209 (-0.346, -0.072)	0.003		
Noise (L_{den})	Lowest quintile			[ref.]	
	Second quintile			-0.069 (-0.214, 0.077)	0.355
	Third quintile			-0.127 (-0.270, 0.015)	0.080
	Fourth quintile			-0.107 (-0.252, 0.039)	0.150
	Highest quintile			-0.254 (-0.395, -0.113)	0.000
Age		-0.028 (-0.034, -0.022)	0.000	-0.028 (-0.033, -0.022)	0.000
Female		-0.096 (-0.182, -0.011)	0.027	-0.123 (-0.206, -0.040)	0.004
Education	Primary/none	[ref.]		[ref.]	
	Secondary	0.216 (0.108, 0.323)	0.000	0.228 (0.121, 0.335)	0.000
	Tertiary/above	0.568 (0.451, 0.685)	0.000	0.596 (0.479, 0.713)	0.000
Employment	Employed	[ref.]			
	Retired	0.001 (-0.126, 0.128)	0.985		
	Other	-0.149 (-0.287, -0.011)	0.035		
Log household income		0.081 (0.023, 0.139)	0.006	0.091 (0.034, 0.149)	0.002
Log residential density				0.082 (0.023, 0.141)	0.007
Physical activity	None	[ref.]		[ref.]	
	Low	0.099 (-0.062, 0.260)	0.227	0.117 (-0.041, 0.275)	0.147
	Moderate	0.124 (-0.036, 0.283)	0.128	0.144 (-0.013, 0.301)	0.072
	High	0.278 (0.098, 0.458)	0.002	0.318 (0.140, 0.495)	0.000
N		1667			1667

Specification (I) includes L_{night} as the noise exposure variable. Specification (II) includes L_{den} as the noise exposure variable.

Noise exposure is categorised using quintiles. ANT score standardised using z-score. Covariates that fail to reject Wald F-test of being jointly equal to zero are omitted from each model. C.I. denotes confidence interval.

Table B2: Sub-sample analysis, average marginal effects of noise exposure (L_{night}) and air pollution exposure, ANT score

		(I) ANT score		(II) ANT score		(III) ANT score		(IV) ANT score	
		dy/dx (95% C.I.)	p-value	*dy/dx* (95% C.I.)	p-value	*dy/dx* (95% C.I.)	p-value	*dy/dx* (95% C.I.)	p-value
Noise	Lowest quintile	[ref.]		[ref.]		[ref.]		[ref.]	
	Second quintile	-0.101 (-0.280, 0.078)	0.266	-0.074 (-0.256, 0.108)	0.423	-0.074 (-0.258, 0.109)	0.426	-0.079 (-0.264, 0.106)	0.400
	Third quintile	-0.379 (-0.626, -0.133)	0.003	-0.319 (-0.562, -0.075)	0.010	-0.329 (-0.577, -0.081)	0.010	-0.320 (-0.571, -0.068)	0.013
	Fourth quintile	-0.033 (-0.256, 0.191)	0.775	0.082 (-0.151, 0.315)	0.489	0.036 (-0.198, 0.269)	0.765	0.049 (-0.187, 0.285)	0.685
	Highest quintile	-0.242 (-0.431, -0.053)	0.012	-0.015 (-0.255, 0.226)	0.905	-0.158 (-0.373, 0.056)	0.147	-0.141 (-0.366, 0.083)	0.216
NO_2	Lowest quintile			[ref.]					
	Second quintile			-0.172 (-0.391, 0.047)	0.124				
	Third quintile			-0.210 (-0.439, 0.018)	0.071				
	Fourth quintile			-0.376 (-0.601, -0.150)	0.001				
	Highest quintile			-0.472 (-0.768, -0.176)	0.002				
$PM_{2.5}$	Lowest quintile					[ref.]			
	Second quintile					-0.126 (-0.351, 0.099)	0.272		
	Third quintile					-0.221 (-0.458, 0.016)	0.068		
	Fourth quintile					-0.304 (-0.561, -0.046)	0.021		
	Highest quintile					-0.226 (-0.551, 0.099)	0.172		
PM_{10}	Lowest quintile							[ref.]	
	Second quintile							-0.116 (-0.346, 0.113)	0.318
	Third quintile							-0.176 (-0.418, 0.065)	0.151
	Fourth quintile							-0.328 (-0.587, -0.068)	0.013
	Highest quintile							-0.245 (-0.575, 0.084)	0.144
N			728		728		728		728

Specifications (I)-(IV) all include L_{night} as the noise exposure variable. In Specification (II), exposure to NO_2 pollution is included. In Specification (III), exposure to $PM_{2.5}$ is included. In Specification (IV), exposure to PM_{10} is included.

Noise exposure and air pollution exposure are categorised using quintiles. ANT score standardised using z-scores. Results correspond to models that adjust for socio-demographic, behavioural and health characteristics. C.I. denotes confidence interval.
